# Isotemporal substitution of accelerometer-derived sedentary behavior and physical activity on physical fitness in young children

**DOI:** 10.1038/s41598-024-64389-7

**Published:** 2024-06-12

**Authors:** Ying Gu, Junghoon Kim, Jiameng Ma, Hongzhi Guo, Hiroko Sano, Ho Jin Chung, Terence Buan Kiong Chua, Michael Yong Hwa Chia, Hyunshik Kim

**Affiliations:** 1 College of Sports Science, Shenyang Normal University, Shenyang, 110034 China; 2 Laboratory of Sports and Exercise Medicine, Korea Maritime & Ocean University, Busan, 49112 Korea; 3 Faculty of Sports Science, Sendai University, Miyagi, 9891693 Japan; 4 National Institute of Education, Nanyang Technological University, Singapore, 637616 Singapore; 5 Graduate School of Human Sciences, Waseda University, Tokorozawa, 3591192 Japan; 6 Kindergardens Teacher Training College, Seitoku University, Tokyo, 108-0073 Japan

**Keywords:** Paediatrics, Public health, Quality of life, Health care

## Abstract

This study investigates the effects of different types of physical activity (PA) on the physical fitness (PF) of young children in Japan, with a particular focus on how substituting sedentary behavior (SB) with active behaviors influences PF. We conducted a cross-sectional analysis of 1843 participants aged 3–6 years from northeastern Japan. Using triaxial accelerometers, we quantified PA, and PF was assessed via standardized tests. The innovative application of isotemporal substitution modeling (ISM) allowed us to analyze the impact of reallocating time from SB to more active states, specifically moderate-to-vigorous physical activity (MVPA) and light physical activity (LPA). Our findings reveal a robust association between increased MVPA and enhanced PF outcomes, underscoring the health benefits of reducing SB. Notably, replacing SB with LPA also showed beneficial effects on certain PF metrics, indicating LPA's potential role in early childhood fitness. These results highlight the critical importance of promoting MVPA and minimizing sedentary periods to bolster PF in young children. The study offers vital insights for shaping public health policies and emphasizes the need to cultivate an active lifestyle from an early age to secure long-term health advantages.

## Introduction

Physical fitness is defined as the ability to perform daily activities with optimal performance, endurance, and strength while managing disease, fatigue, stress, and reduced sedentary behavior^1^. Physical fitness has multiple components (Cardiovascular endurance, muscular strength, muscular endurance, flexibility, body composition) and is conceptualized as either performance- or health-related^2^. High levels of physical fitness in early childhood are reported to be important because they can help maintain and improve health in adulthood^3^. Despite its importance, previous studies have reported a global decline in young children fitness over the past few decades^4,5^. It is negatively associated with cardiometabolic disorders, body composition, poorer cognitive control, and poorer academic performance, and is recognized as a global early childhood health issue due to these negative changes in PF^6–9^. Previous studies have shown that physical activity (PA) in young children is positively associated with PF^10,11^. Increasing moderate to vigorous physical activity (MVPA) can significantly improve PF in physically inactive young children^12^. The World Health Organization (WHO) published the "WHO 2020 Guidelines on Physical Activity and Sedentary Behaviour" and aims to reduce time spent in SB and increase PA for people of all ages as a goal of the Global Action Plan^13^. SB is defined as any awake behavior characterized by an energy expenditure of ≤ 1.5 metabolic equivalents (METs) while sitting, lying, or reclining^14^, and young children report spending more than 60% of their day sedentary^15^. Furthermore, there is evidence that habitual SB in infancy persists throughout childhood and into adulthood^16^, suggesting that we should spend as much time as possible in PA during waking hours for health benefits such as improved PF^17^.

From infants to the elderly, human behavior is interdependent, and in terms of kinematics, daily activities during waking hours are composed of SB, LPA, and MVPA. There are only so many hours in the day that individuals have at their disposal, and they must reduce the time they spend on one activity to make room for another. For example, increasing MVPA time by 30 min during waking hours requires a corresponding decrease in time for other activities such as SB and LPA, implying a substitution of interdependent activities. Thus, a statistical method that can estimate the association of substitute time spent on an activity while holding the total time of the day constant is an isotemporal substitution modeling (ISM) analysis^18^. Previous research using ISMs has shown that they can improve our understanding of the relationships between different physical activities and their associations with health-related factors^19–22^, and help shape public health guidelines and promotion strategies^23^. Research on children under 6 years of age shows that physical activity is associated with health benefits such as reduced obesity, improved bone and cardiovascular health, and improved cognition^24^. Most public health guidelines for PA in young children recommend participation in some amount of MVPA, but there is a lack of evidence that SB and LPA can replace MVPA.

In a typical 24-h day, an infant's behavior is composed of sleep (~ 40%), SB (~ 40%), LPA (~ 15%), and MVPA (< 5%)^25^, and the age-related trajectory of PA suggests that most of the increase in SB comes from the loss of LPA, with time spent in MVPA progressively shifted to SB rather than LPA^26^. As such, increasing SB will decrease most LPA, warranting a review of LPA by ISM. In addition, the studies of ISM reported so far have reported on obesity, cardiovascular metabolism, and physical fitness in Japanese young children^27–30^. Therefore, a review of the interdependencies of SB, LPA, and MVPA within the finite hours of the day is particularly important. The present research was a cross-sectional study of Japanese young children used to investigate the effects of behavioral changes between SB and various PAs on PF. We hypothesized that statistically replacing the same amount of SB with LPA, MVPA would Improving PF of young children.

## Materials and methods

### Study design and participants

This is a cross-sectional study using data from 2019 to 2022 from “the Eat Well, Be Active, Sleep Well” study, a project aimed at improving the 24-h lifestyle of Japanese young children^31,32^. We selected Ohira-Mura (population 5918, area 60.32 km^2^) and Tomiya-City (population 52,340, area 49.18 km^2^) in northeastern Japan as the target areas and measured the PA of young children using accelerometers. These children were recruited from five kindergartens in the two areas and measured over four years. After explaining the purpose of the study in accordance with the Declaration of Helsinki, we obtained signed informed consent from the young children (aged 3–6 years), their parents, and teachers, and analyzed data from 1843 participants (52.4% boys, 47.6% girls; data collection rate = 65.8%). This study was approved by the Ethics Committee of the university (SU29-22, SU03-05).

### Measures

#### Accelerometer measures of physical activity and sedentary behaviors

PA was measured using a triaxial accelerometer (Active Style Pro HJA-750C, Omron Health Care Co. Ltd., Tokyo, Japan). The accelerometer was worn on the left (or right) side of the waist, relative to the belly button, for one week from waking to bedtime (typically from 7:00 a.m. to 9:00 p.m.), except when showering or swimming. In addition, we extracted data only from participants who wore the accelerometer at least 4 days per week and for more than 10 h per day to measure daily PA^33^. The Active Style Pro HJA-750C estimates metabolic equivalents (METs) every 10 or 60 s, based on the combined accelerations, measured by an internal tri-axial accelerometer (60-s epoch selected in this study). The accelerometer records anteroposterior (x-axis), mediolateral (y-axis), and vertical (z-axis) accelerations with a resolution of 3 mG at 32 Hz, and has the ability to classify physical activity into the locomotive and sedentary activities. The accelerometer directly predicts the METs without the need of any additional process, using a multiple regression model, which is based on 12 key activities (7 locomotive activities and 5 household activities). This proprietary algorithm is able to distinguish the locomotive and household activity by the process filtered and unfiltered acceleration data^34^. The Active Style Pro used in this study provided Metabolic Equivalent Task (MET) values derived from an adult prediction equation. However, since it has been reported that MET values are overestimated in young children compared to adults, we analyzed them using a conversion formula obtained from a previous study^35,36^. Where is children’s METs, t is the output (METs) classified into “non-locomotive” by the Active style Pro HJA-750C. The point of intersection between the equation for light or higher intensity activities and the equation for sedentary, that is 1.9 METs, based on the relationship between synthetic acceleration and METs in the Active style Pro HJA-750C.

Equation for non-locomotive activity METs

$${\text{METs}} = 0.0103 \times (({\text{X}}{-}0.8823)/0.0351) + 0.9\;(X\;is\;lessthan\;1.9\;METs).$$

$${\text{METs}} = 0.0103 \times (({\text{X}}-1.3435)/0.0196) + 0.9({\text{X}}\;is\;2.0\;{\text{Mets}}\;{\text{or}}\;{\text{more}})$$

Equation for locomotive activity METs

$${\text{METs}} = 1.0012 + 0.0037 \times ({\text{X}}{-}1.1128) \, / \, 0.0086$$

#### Physical fitness

This PF test is a national project in Japan, using items tested on young children in 21 cities across the country over a three-year period from 2007 to 2009. PF was assessed using four measures of basic PF among the eight measures of the Japanese Ministry of Education, Culture, Sports, Science, and Technology^37^. The physical fitness test consisted of four components: 25-m sprint (speed of body movement), standing long jump (lower body explosive strength), tennis ball throw (upper body explosive strength), and handgrip strength. The PF tests were administered by the researcher with the help of one teacher from each preschool. After thorough training on how to measure PF, at least two teachers participated in each measure.

The 25- meter sprint was assessed on a 30-m straight track. The preschoolers were asked to run at full speed. The distance from the starting point to the 25-m point was measured, and the time was recorded in 0.1-s increments with a stopwatch. The standing long jump test was performed as follows. The preschoolers were asked to jump using the recoil of the arms, body, and legs after standing on a starting plate. The distance from the marked start line to the landing point of the nearest heel was measured at 90 degrees to the marked line. The distance was recorded in 0.1 cm increments, and the best record after two attempts was used for the analysis. The standing long jump test evaluates the explosive strength of the lower extremities. The best of the two attempts was recorded in centimeters (cm). The tennis ball throw test was conducted as follows. Preschooler threw a tennis ball as far as possible without running after standing and without stepping on the line with the front foot. If the preschooler stepped on the line or the ball was thrown out of bounds, the result was not recorded. The best of two attempts was used for analysis. Results below 100 cm were not recorded, and the throwing distance was measured in centimeter increments.

The Takei digital dynamometer, model T.K.K.5401, was used to record the maximum value (kilograms) of the handgrip strength. The dimensions of the unit are approximately 154 (weight) 235 (diameter) 62 (height) mm. The weight is about 0.63 kg. With a liquid crystal display, it has the measuring range of 5.0–100 kg. The Japanese Ministry of Education, Culture, Sports, Science, and Technology has specified the details of the measuring instruments and methods for all measurement items^38^ and all tests were conducted in full compliance with these specifications.

#### Body mass index and anthropometric measurements

To examine the children' body mass index (BMI), height and body mass were measured in increments of 0.1 cm and 0.1 kg, respectively, and objective measurements were taken by researchers. BMI z-scores were calculated according to WHO growth standards^39^. For children younger than 5 years, overweight and obesity were categorized as a BMI z-score of 2 standard deviations and 3 standard deviations or more, respectively^40^. Children' BMI percentiles were categorized according to the age criteria in the U.S. Centers for Disease Control and Prevention growth charts as follows: Obese (BMI percentile ≥ 95), Overweight (85 ≤ BMI percentile < 95), Healthy weight (5 ≤ BMI percentile < 85), and Underweight (BMI percentile < 5)^41^. This model development process led to a final model for the prediction of natural logarithm of fat-free mass (and subsequently for fat mass = weight − exp (prediction of natural logarithm of fat-free mass)) based on the selected predictors along with their corresponding estimated β coefficients and the associated intercept term. exp = exponential function, ln = natural logarithmic transformation. Score 1 if child is of black (BA), south Asian (SA), other Asian (AO), or other (other) ethnic origins and score 0 if not. If child is of unknown ethnic group, treat as of white ethnic origins. Height is measured in metres, body mass in kilograms, age in years, and fat mass in kilograms. In addition, body fat (FM) was calculated using the following formula^42,43^:

$${\text{FM = eight}}{-}{\text{exp}}(0.{3}0{73} \times {\text{height}}^{{2}} - {1}0.0{155} \times {\text{weight}}^{{ - {1}}} + 0.00{4571} \times {\text{weight}} - 0.{918}0 \times {\text{ln}}\left( {{\text{age}}} \right) + 0.{6488} \times {\text{age}}^{{0.{5}}} + 0.0{4723} \times {\text{male}}\left( {{\text{female}}} \right) + {2}.{8}0{55})$$

### Statistical analysis

Study participant characteristics, such as age and BMI, were expressed as percentages (%) and analyzed for statistical significance by gender using the chi-squared test. Independent t-tests were used to estimate means and standard deviations (SDs) for continuous variables (BMI z-score, FM, accelerometer activity, and PF) by gender. To assess the impact of SB, we developed three different logistic regression models for each activity type on PF tests (25-m sprint, standing long jump, tennis ball throw, and handgrip strength). Odds ratios (ORs) and confidence intervals (CIs) were calculated for PF as a function of time spent active every 30 min. First, we developed a single factor model to assess the individual associations of SB, LPA, and MVPA after adjusting for age, sex, BMI, and total accelerometer wear time. This model shows the overall effect of the behavioral variables of SB, LPA, and MVPA. However, because the behavioral variables were not corrected for each other, it does not necessarily mean that the independent effects were verified. Second, we developed partition models to examine independent associations for each type of activity. We included covariates for SB, PA (time spent in LPA and MVPA), and total accelerometer wear time in one model. In the partition model, SB, LPA, and MVPA are all included in the regression model, and confounding variables are also included. This model examines the independent effects of each behavioral variable. As a cautionary note for the partition model, it is necessary to check for multicollinearity due to high correlations among behavioral variables. Finally, we used the ISM to estimate the association between replacing 30 min per day of time spent in one activity with the same amount of time spent in another activity and total accelerometer wear time with PF. The IS model can be said to show the substitution effect when one action is substituted for an equal amount of another action. This is a unique feature of the IS model, as it suggests that the relationship between physical activity and health outcomes, for which no consistent trend was observed in the previous model, can be more clearly shown. For example, to assess the effect of replacing 30 min of SB with LPA and MVPA, we removed sedentary time from the overall model, which included other types of activity and total wear time, after adjusting for covariates. In this case, the OR of MVPA represents the effect of replacing 30 min of sedentary time per day with MVPA, while holding other activity variables and total wear time constant.

All statistical analyses were conducted using IBM SPSS 26.0 (IBM, Armonk, NY, USA), with a significance level set at p < 0.05.

### Ethics approval and consent to participate

The study was conducted in accordance with the guidelines of the Declaration of Helsinki and was approved by the Institutional Review Board of the Sendai University Ethics Committee of the Faculty of Sports Science (IRB number: SU2019-31, SU03-05). Considering the involvement of minor participants in this study, informed consent was obtained from their parents and/or legal guardians.

## Results

Table 1 shows the general characteristics of the participants by sex. The mean age was 4.3 ± 0.9 years, the mean BMI z-score was 0.2 ± 0.02 SEM, indicating a significant deviation from zero, which is caused by overweight children, and the mean BF percentage was 2.5 ± 1.4 kg. Total accelerometer wear time, SB, LPA, and MVPA were 734.8 ± 105.1 min/day, 435.4 ± 96.0 min/day, 251.2 ± 38.5 min/day, and 48.2 ± 15.7 min/day, respectively. SB and LPA were longer in girls than in boys. However, the time spent on MVPA was significantly longer in boys than in girls (p < 0.001). In addition, among the PF items, the 25-m sprint, standing long jump, tennis ball throwing, and handgrip were 7.4 ± 1.7 s, 92.0 ± 24.8 cm, 5.3 ± 2.9 m, and 12.5 ± 4.0 kg, respectively. Boys performed significantly better than girls on all PF measures.

**Table 1:** Characteristics of the participants.

	Total (n = 1843)	Boys (n = 966)	Girls (n = 877)	*p*-value
Age (years: mean, SD)	4.3 ± 0.9	4.3 ± 0.9	4.3 ± 1.0	0.684
3	428 (23.2%)	224 (23.3%)	204 (23.3%)	0.917
4	613 (33.3%)	326 (33.7%)	287 (32.7%)	
5	607 (32.9%)	318 (32.9%)	289 (33.0%)	
6	195 (10.6%)	98 (10.1%)	97 (11.0%)	
Height (cm: mean, SD)	105.1 ± 7.3	105.6 ± 7.2	104.7 ± 7.4	0.010
Body mass (kg: mean, SD)	17.4 ± 3.0	17.6 ± 3.0	17.2 ± 3.0	0.003
BMI z-score (kg/m^2^, mean, SEM)^a^	0.22 ± 0.02	0.27 ± 0.03	0.17 ± 0.03	0.028
BMI percentile (%)^b^				
Underweight	30 (1.6%)	16 (1.7%)	14 (1.6%)	0.016
Normal weight	1506 (82.5%)	764 (79.9%)	742 (85.3%)	
Overweight	198 (10.8%)	117 (12.2%)	81 (9.3%)	
Obesity	92 (5.1%)	59 (6.2%)	33 (3.8%)	
Fat mass (kg)	2.5 ± 1.4	2.8 ± 1.4	2.1 ± 1.4	< 0.001
Accelerometer-derived MB (mean, SD; min/d)				
Accelerometer wear-time (min)	734.8 ± 105.1	736.1 ± 106.9	733.5 ± 103.4	0.664
Sedentary behavior (min)	435.4 ± 96.0	429.7 ± 95.2	441.3 ± 91.5	0.032
Light intensity physical activity (min)	251.2 ± 38.5	254.2 ± 39.4	248.0 ± 37.3	0.004
MVPA (min)	48.2 ± 15.7	52.1 ± 16.3	44.1 ± 13.8	< 0.001
Number of steps (steps)	9063 ± 2460	9547 ± 2614	8548 ± 2171	< 0.001
Physical fitness (mean, SD)				
25 m sprint (s)	7.4 ± 1.7	7.3 ± 1.6	7.5 ± 1.7	0.004
Standing long jump (cm)	92.0 ± 24.8	93.6 ± 26.0	90.1 ± 23.2	0.002
Ball throw (m)	5.3 ± 2.9	6.2 ± 3.3	4.4 ± 2.0	< 0.001
Handgrip strength (kgf)	12.5 ± 4.0	12.8 ± 4.1	12.2 ± 3.9	0.005

*SD* standard deviation, *BMI* body mass index, *MB* movement behaviors, *MVPA* moderate to vigorous physical activity. P-values were calculated using t-test for continuous variables and chi-square test for categorical variables.

^a^BMI z-scores were calculated according to the World Health Organization (WHO) growth standards^33^.

^b^Children' BMI percentiles were categorized according to the age criteria in the U.S. Centers for Disease Control and Prevention growth charts^34^.

Table 2 presents the single factor model, partition model, and ISM of the associations between SB, LPA, and MVPA and PF outcomes. After adjustment for covariates in the single factor model, only SB and MVPA were significantly associated with the 25-m sprint and standing long jump. In addition, only MVPA was associated with handgrip strength and tennis ball throw. In the partition model, among the PF items, boys and girls were significantly associated with MVPA in handgrip strength, the 25-m sprint, and tennis ball throw, and only girls were associated with SB in handgrip strength. In the ISM, replacing SB with MVPA was associated with a positive effect between PF outcomes, and replacing LPA with MVPA also had a positive effect on these outcomes.

**Table 2:** Single factor, partition, and isotemporal substitution models examining the association of SB, LPA, and MVPA with 25 m sprint, standing long jump, ball throw and handgrip strength.

	Boys			Girls		
	SB	LPA	MVPA	SB	LPA	MVPA
	*β* (95%CI)	*β* (95%CI)	*β* (95%CI)	*β* (95%CI)	*β* (95%CI)	*β* (95%CI)
25 m sprint						
Single models	0.06 (0.01, 0.12)	− 0.03 (− 0.12, 0.06)	− 0.39 (− 0.57, − 0.21)	0.06 (0.01, 0.15)	− 0.03 (− 0.09, 0.06)	− 0.63 (− 0.84, − 0.42)
Partition models	0.01 (− 0.03, 0.03)	− 0.01 (− 0.03, 0.03)	− 0.51 (− 0.72, − 0.30)	− 0.03 (− 0.06, 0.01)	− 0.01 (− 0.03, 0.03)	− 0.87 (− 1.14, − 0.63)
IS models						
Replace SB	Dropped	0.06 (− 0.01, 0.12)	0.48 (0.30, 0.69)	Dropped	0.09 (− 0.18, 0.01)	0.87 (0.60, 1.11)
Replace LPA	− 0.06 (− 0.12, 0.01)	Dropped	0.60 (0.33, 0.84)	− 0.09 (− 0.01, 0.18)	Dropped	1.02 (0.84, 1.32)
Replace MVPA	− 0.48 (− 0.69, − 0.30)	− 0.60 (− 0.84, − 0.33)	Dropped	− 0.87 (− 1.11, − 0.63)	− 1.02 (− 1.32, − 0.84)	Dropped
Standing long jump						
Single models	− 1.62 (− 2.61, − 0.63)	0.90 (− 0.39, 2.19)	8.58 (5.85, 11.31)	− 0.69 (− 1.65, 0.27)	0.03 (− 1.17, 1.26)	6.69 (3.66, 9.69)
Partition models	0.48 (− 0.01, 0.96)	1.14 (2.67, − 2.46)	10.89 (7.62, 14.19)	0.18 (− 0.24, 0.63)	− 1.80 (1.21, − 3.09)	9.54 (5.07, 13.17)
IS models						
Replace SB	Dropped	− 1.62 (− 3.27, 0.03)	− 10.41 (− 13.62, − 7.20)	Dropped	− 1.98 (− 3.99, − 0.06)	− 9.36 (− 12.90, − 5.79)
Replace LPA	1.62 (− 0.03, 3.27)	Dropped	− 12.03 (− 16.20, − 7.86)	1.98 (− 0.06, 3.99)	Dropped	− 10.11 (− 15.78, − 6.84)
Replace MVPA	10.41 (7.20, 13.62)	12.03 (7.86, 16.20)	Dropped	9.36 (5.79, 12.90)	10.11 (6.84, 15.78)	Dropped
Ball throw						
Single models	− 0.21 (− 0.33, − 0.09)	0.15 (− 0.03, 0.30)	1.02 (0.66, 1.41)	− 0.18 (− 0.27, − 0.09)	0.18 (− 0.03, 0.39)	0.69 (0.45, 0.96)
Partition models	0.01 (− 0.06, 0.06)	− 0.15 (− 0.33, 0.03)	1.20 (0.75, 1.65)	− 0.03 (− 0.06, 0.03)	0.01 (− 0.12, 0.12)	0.66 (0.33, 0.96)
IS models						
Replace SB	Dropped	− 0.15 (− 0.36, 0.06)	− 1.20 (− 1.62, − 0.75)	Dropped	− 0.03 (− 0.15, 0.09)	− 0.66 (− 0.99, − 0.36)
Replace LPA	0.15 (− 0.06, 0.36)	Dropped	− 1.35 (− 1.92, − 0.78)	0.03 (− 0.09, 0.15)	Dropped	− 0.63 (− 1.05, − 0.24)
Replace MVPA	1.20 (0.75, 1.62)	1.35 (0.78, 1.92)	Dropped	0.66 (0.15, 0.99)	0.63 (0.24, 1.05)	Dropped
Handgrip strength						
Single models	− 0.12 (− 0.30, 0.06)	− 0.03 (− 0.27, 0.21)	1.11 (0.60, 1.62)	− 0.12 (− 0.33, 0.06)	0.06 (− 0.21, 0.30)	1.05 (0.42, 1.68)
Partition models	0.03 (− 0.06, 0.12)	− 0.18 (− 0.27, 0.09)	1.59 (0.96, 2.22)	− 0.06 (− 0.15, 0.03)	− 0.21 (− 0.33, 0.18)	1.35 (0.57, 2.13)
IS models						
Replace SB	Dropped	− 0.39 (− 0.90, 0.12)	− 1.59 (− 2.19, − 0.96)	Dropped	− 0.24 (− 0.48, 0.06)	− 1.41 (− 2.16, − 0.63)
Replace LPA	0.39 (− 0.12, 0.90)	Dropped	− 1.98 (− 2.76, − 1.20)	0.24 (− 0.06, 0.48)	Dropped	− 1.65 (− 2.61, − 0.69)
Replace MVPA	1.59 (0.96, 2.19)	1.98 (1.20, 2.76)	Dropped	1.41 (0.63, 2.16)	1.65 (0.69, 2.61)	Dropped

Single model: adjusted for age, BMI z-score and accelerometer wear-time. Partition model: adjusted for single model covariates plus other types of activities without accelerometer wear-time. IS model: dropped one activity from the full model including other types of activities and total ware time after adjusting for covariates.

*SB* sedentary behaviour, *LPA* light physical activity, *MVPA* moderate-to-vigorous physical activity, *CI* confidence interval, *IS model* isotemporal substitution model.

**Figure 1:**
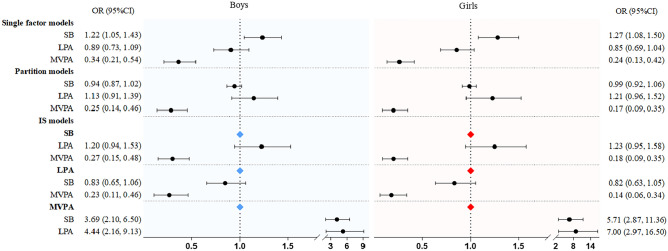
Results of single factor, partition, and IS models examining the association of sedentary behavior and MVPA with 25 m sprint by boys and girls. *Note* Values were odds ratio (95%CI), *OR* odds ratio, *CI* confidence interval.

We examined all analyses as a sensitivity analysis using models stratified by sex (boys or girls). Figure 1 shows the results of the single factor model, partition model, and ISM for associations with 25 m sprint outcomes by sex. Similar results were found for girls as for boys, with a strong association with MVPA in the single, partition, and IS models.

**Figure 2:**
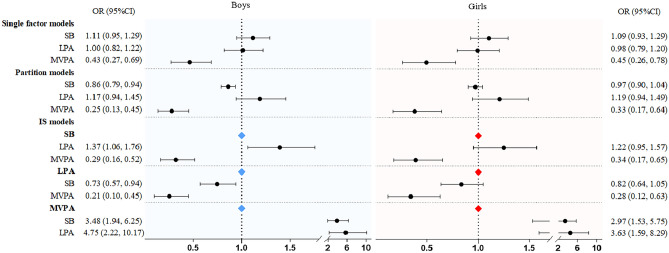
Results of single factor, partition, and IS models examining the association of sedentary behavior and MVPA with standing long jump by boys and girls. Values were odds ratio (95%CI), *OR* odds ratio, *CI* confidence interval.

Figure 2 shows the results of the single factor model, partition model, and ISM for associations with long jump outcomes by sex. Similar results were found for girls as for boys, with a strong association with MVPA in the single, partition, and IS models. However, for boys, an association with SB was found in the partition and IS models.

**Figure 3:**
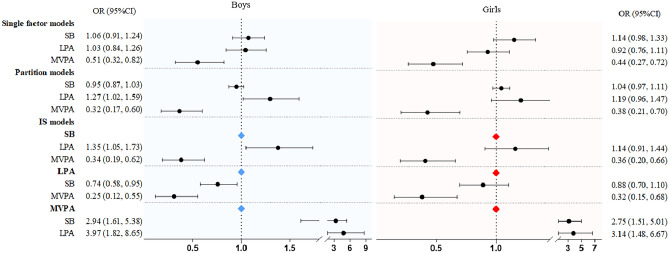
Results of single factor, partition, and IS models examining the association of sedentary behavior and MVPA with ball throw by boys and girls. Values were odds ratio (95%CI), *OR* odds ratio, *CI* confidence interval.

Figure 3 shows the results of each model for associations with ball throw outcomes by sex. Similar results were found for girls as for boys, with a strong association with MVPA in the single, partition, and IS models. However, in boys, association with LPA was found in the partition, and IS models.

**Figure 4:**
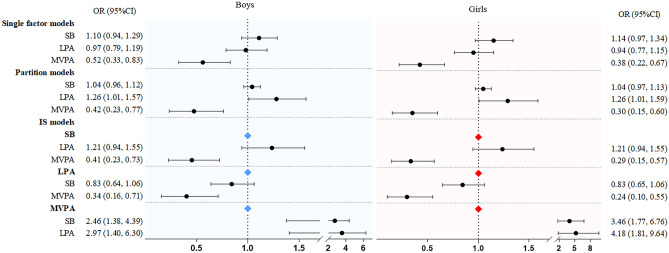
Results of single factor, partition, and IS models examining the association of sedentary behavior and MVPA with handgrip strength by boys and girls. Values were odds ratio (95%CI), *OR* odds ratio, *CI* confidence interval.

Figure 4 shows the results of the single factor model, partition model, and ISM for associations with handgrip strength outcomes by sex. Similar results were found for girls as for boys, with a strong association with MVPA in the single, partition, and IS models.

## Discussion

In a cross-sectional study of Japanese young children, we examined the association of substituting accelerometer-measured SB for LPA or MVPA with beneficial effects on PF. Our study is apparently the first to describe the associations between accelerometer-measured SB, PA, and PF in a large representative sample of Japanese preschoolers.

Our results are consistent with many previous studies, and it appears that meaningful effects on PF promotion can be obtained from MVPA, which is also supported by systematic reviews in young children showing associations between MVPA and several health indicators, including PF^44–47^. Previous studies of the 40-year period from 1966 to 2002 have shown a stagnation in the improvement of PF until 1986, a significant decline in PF from 1986 to 1997^48^, and a slight slowing of the decline from 1997 to 2002^49^. In addition, a trend that remained almost unchanged from 2002 to 2019 was confirmed, but 6) after the COVID-19, it decreased significantly again^50^. Our main finding was that, using a method based on ISM^18^, we found that replacing 30 min/day of SB with MVPA may contribute to optimal fitness gains in Japanese young children. An important issue to consider in the ISM is the timing of substitution, and LPA accounts for the majority of PA in daily life; therefore, 30 min of PA of varying intensities was used to substitute for SB, as it is relatively easy to substitute SB with 30 or 60 min of LPA^44,51^. While much of the literature focuses on the negative effects of prolonged SB^52^, our results suggest that it would be more appropriate to shift the focus to the importance of reallocating time spent on SB to time spent on MVPA. In both single factor and partition models, we found independent significant associations between MVPA and low PF. Our findings support previous research on the importance of PA in promoting PF in preschool children^53^. These findings suggest that encouraging young children to increase MVPA and reduce sedentary time may have beneficial effects on health-related PF. Therefore, it is also important to understand whether the association between MVPA and SB is independent across PF levels.

Our next important finding is the positive effect of replacing SB with LPA on some PT items. Previous studies have reported associations of LPA with PF, such as handgrip strength and leg strength^54,55^. Furthermore, the age-related trajectory of PA suggests that most of the increase in SB is due to the loss of LPA, and that time spent in MVPA is progressively transferred to SB rather than LPA, so more attention should be paid to increasing LPA^56^. LPA may serve as a prerequisite for participation in MVPA, and increasing LPA levels may be a viable gateway to improving overall daily PA. In particular, LPA engagement is more important for young children with low levels of MVPA^57^. To promote LPA engagement, parents should, for example, reduce restrictions on active play due to overprotection, encourage walking to school, and promote participation in PA of all intensities, including participation in recess activity programs.

This study highlighted the importance of increasing the amount of LPA and MVPA in a child's day to improve PF. This information can provide important guidance for the selection of subjects and measurement variables for future intervention studies. In particular, it is suggested that preschool teachers or caregivers should pay close attention to monitoring the ratio of LPA to MVPA when planning PA and try to increase the amount of MVPA appropriately. As previous studies have reported that toddlers who participated in structured play had higher PA levels than those who participated in unstructured play, we suggest that future intervention studies in preschoolers should focus on increasing PA through structured play in general and consequently improving PF levels^58,59^. Our study highlighted the potential of ISM in providing more flexible options for young children to change their sedentary lifestyles and establish daily PA habits. A systematic review found that parental engagement in PA, rather than environmental factors, is an important determinant of successful interventions for young children. Because young children spend the majority of their waking hours in preschool or childcare, they are less likely to be engaging in appropriate amounts of PA on weekends than on weekdays^60–62^. Therefore, we suggest that it may be important for parents to engage in regular outdoor activities in nature or in family PA on weekends in order to develop or promote PF in their children.

Our study has several limitations. First, although accelerometers have the advantage of objectively measuring PA, they may have limitations in representing certain types of PA, such as overestimating behaviors such as standing or lying down as LPA or SB. Second, the cross-sectional design of the present study may limit the causal hypotheses between SB, different PA, and health-related PF. Therefore, future longitudinal or intervention studies should examine the direction of causal effects of actual time to change each behavior on health-related PF. Finally, because this study was conducted among young children in northeastern Japan, our findings may not be generalizable to all preschool children in Japan. This is especially important for rural and low-income preschoolers, who have been reported to be more likely to have delayed health-related PF due to a lack of PA facilities and exercise tools, and difficulty participating in contextualized PA (e.g., running, jumping, throwing, catching, etc.) such as local sports clubs^63,64^. Therefore, further studies with samples from different regions should be conducted.

## Conclusions

Our study found that replacing SB with equal hours of MVPA using the ISM was associated with improved PF in Japanese young children. Also, next finding is that we found a positive effect of replacing SB with LPA on some PT items (handgrip strength, 25-m sprint, standing long jump, and tennis ball throw). Our findings suggest that in the future, replacing SB with LPA and MVPA within the same time period may be useful in designing tailored intervention studies that are effective in improving PF (standing long jump, ball throwing, and handgrip strength) in Japanese young children.

## Data Availability

The datasets used and/or analysed during the current study available from the corresponding author on reasonable request.

## References

[1] Campbell, N., Jesus, S. & Prapavessis, H. Physical fitness. *Encyclopedia of Behavioral Medicine* 1486–1489 (2013).

[2] Ruiz JR (2009). Predictive validity of health-related fitness in youth: A systematic review. Br. J. Sports Med..

[3] Ortega FB (2018). Fitness and fatness as health markers through the lifespan: An overview of current knowledge. Prog. Prev. Med..

[4] Morales-Demori R, Jamil O, Serratto M (2017). Trend of endurance level among healthy inner-city children and adolescents over three decades. Pediatr. Cardiol..

[5] Tomkinson GR, Olds TS (2007). Secular changes in pediatric aerobic fitness test performance: The global picture. J. Diabetes Res..

[6] Andersen LB (2015). A new approach to define and diagnose cardiometabolic disorder in children. J. Diabetes Res..

[7] Ornelas RT, Silva AM, Minderico CS, Sardinha LB (2011). Changes in cardiorespiratory fitness predict changes in body composition from childhood to adolescence: Findings from the European youth heart study. Phys. Sportsmed..

[8] Pontifex MB (2014). The differential association of adiposity and fitness with cognitive control in preadolescent children. Monogr. Soc. Res..

[9] Sardinha LB (2016). Longitudinal relationship between cardiorespiratory fitness and academic achievement. Med. Sci. Sports Exerc..

[10] Bürgi F (2011). Relationship of physical activity with motor skills, aerobic fitness and body fat in preschool children: A cross-sectional and longitudinal study (Ballabeina). Int. J. Obes..

[11] Leppänen MH (2016). Physical activity intensity, sedentary behavior, body composition and physical fitness in 4-year-old children: Results from the ministop trial. Int. J. Obes..

[12] Janssen I, LeBlanc AG (2010). Systematic review of the health benefits of physical activity and fitness in school-aged children and Youth. Int. J. Behav. Nutr. Phys. Act..

[13] Ding D (2020). Physical activity guidelines 2020: Comprehensive and inclusive recommendations to activate populations. Lancet.

[14] Tremblay MS (2017). Sedentary behavior research network (SBRN): Terminology consensus project process and outcome. Int. J. Behav. Nutr. Phys. Act..

[15] Baptista F (2012). Prevalence of the Portuguese population attaining sufficient physical activity. Med. Sci. Sports Exerc..

[16] Biddle SJH, Pearson N, Ross GM, Braithwaite R (2010). Tracking of sedentary behaviours of young people: A systematic review. Prev. Med..

[17] World Health Organization. *Guidelines on Physical Activity, Sedentary Behaviour and Sleep for Children Under 5 Years of Age*. https://www.who.int/publications/i/item/9789241550536 (2019). 31091057

[18] Mekary RA, Willett WC, Hu FB, Ding EL (2009). Isotemporal substitution paradigm for physical activity epidemiology and weight change. Am. J. Epidemiol..

[19] Van Der Velde JH (2017). Sedentary behavior, physical activity, and fitness—the maastricht study. Med. Sci. Sports Exerc..

[20] Ekblom-Bak E (2016). Scapis Pilot Study: Sitness, fitness and fatness—is sedentary time substitution by physical activity equally important for everyone’s markers of glucose regulation?. J. Phys. Act Health.

[21] Ma J, Kim H, Kim J (2021). Isotemporal substitution analysis of accelerometer-derived sedentary behavior and physical activity on cardiometabolic health in Korean adults: A population-based cross-sectional study. Int. J. Environ. Res. Public Health.

[22] Ma J, Ma D, Kim J, Wang Q, Kim H (2021). Effects of substituting types of physical activity on body fat mass and work efficiency among workers. Int. J. Environ. Res. Public Health.

[23] Tremblay MS (2016). Canadian 24-hour movement guidelines for children and youth: An integration of physical activity, sedentary behaviour, and sleep. Appl. Physiol. Nutr. Metab..

[24] Pate RR (2019). Physical activity and health in children younger than 6 years: A systematic review. Med. Sci. Sports Exerc..

[25] Chaput J-P, Carson V, Gray C, Tremblay M (2014). Importance of all movement behaviors in a 24 hour period for overall health. Int. J. Environ. Res. Public Health.

[26] Knaeps S (2016). Ten-year change in sedentary behaviour, moderate-to-vigorous physical activity, cardiorespiratory fitness and cardiometabolic risk: Independent associations and mediation analysis. Br. J. Sports Med..

[27] Smith E, Fazeli F, Wilkinson K, Clark CC (2020). Physical behaviors and fundamental movement skills in British and Iranian children: An isotemporal substitution analysis. Scand. J. Med. Sci. Sports.

[28] Dooley EE (2020). Adiposity, cardiovascular, and health-related quality of life indicators and the reallocation of waking movement behaviors in preschool children with overweight and obesity: An isotemporal data analysis. PLoS ONE.

[29] Huang WY, Wong SHS, He G, Salmon J (2016). Isotemporal substitution analysis for sedentary behavior and body mass index. Med. Sci. Sports Exerc..

[30] Bezerra TA (2020). 24-hour movement behaviour and executive function in preschoolers: A compositional and isotemporal reallocation analysis. Eur. J. Sport Sci..

[31] Hyunshik K, Jiameng M, Sunkyoung L, Ying G (2021). Change in Japanese children’s 24-hour movement guidelines and mental health during the COVID-19 pandemic. Sci. Rep..

[32] Kim H, Ma J, Kim J, Xu D, Lee S (2021). Changes in adherence to the 24-hour movement guidelines and overweight and obesity among children in northeastern Japan: A longitudinal study before and during the COVID-19 pandemic. Obesities.

[33] Trost SG, Pate RR, Freedson PS, Sallis JF, Taylor WC (2000). Using objective physical activity measures with youth: How many days of monitoring are needed?. Med. Sci. Sports Exerc..

[34] Japan Physical Activity Research Platform. *Japan Physical Activity Research Platform*. http://paplatform.umin.jp (2020) (**in Japanese**).

[35] Oshima Y (2010). Classifying household and locomotive activities using a triaxial accelerometer. Gait Posture.

[36] National Institutes of Health. *SAS Programs for Analyzing NHANES 2003–2004 Accelerometer Data*. https://epi.grants.cancer.gov/nhanes-pam/ (2021).

[37] Ministry of Education, Culture, Sports, Science and Technology-Japan. *Early Childhood Movement*. https://www.mext.go.jp/a_menu/sports/undousisin/index.htm (2012) (**in Japanese**).

[38] Ministry of Education, Culture, Sports, Science and Technology-Japan. *The Early Childhood Exercise Guidelines*. https://www.mext.go.jp/a_menu/sports/undousisin/1319772.htm (2012) (**in Japanese**).

[39] De Onis M (2006). Who child growth standards based on length/height, weight and age. Acta Paediatr..

[40] De Onis M, Lobstein T (2010). Defining obesity risk status in the general childhood population: Which cut-offs should we use?. Int. J. Pediatr. Obese..

[41] Barlow SE (2007). Expert committee recommendations regarding the prevention, assessment, and treatment of child and adolescent overweight and Obesity: Summary Report. Pediatrics.

[42] Hudda MT (2019). Development and validation of a prediction model for fat mass in children and adolescents: Meta-analysis using individual participant data. BMJ.

[43] Wang Q, Guo H, Chen S, Ma J, Kim H (2022). The Association of body mass index and fat mass with health-related physical fitness among Chinese schoolchildren: A study using a predictive model. Int. J. Environ. Res. Public Health.

[44] Santos DA, Marques A, Minderico CS, Ekelund U, Sardinha LB (2017). A cross-sectional and prospective analyse of reallocating sedentary time to physical activity on children’s cardiorespiratory fitness. J. Sports Sci..

[45] Jones MA (2019). Associations of accelerometer-measured sedentary time, sedentary bouts, and physical activity with adiposity and fitness in children. J. Sports Sci..

[46] Jaakkola T, Yli-Piipari S, Huotari P, Watt A, Liukkonen J (2016). Fundamental movement skills and physical fitness as predictors of physical activity: A 6-year follow-up study. Scand. J. Med. Sci. Sports.

[47] Lima RA (2017). Physical activity and motor competence present a positive reciprocal longitudinal relationship across childhood and early adolescence. J. Phys. Act. Health.

[48] Sugihara T, Kondo M, Mori S, Yoshida I (2006). Chronological change in preschool children’s motor ability development in Japan from the 1960s to the 2000s. Int. J. Sport Health Sci..

[49] Mori S, Sugihara T, Yoshida I (2010). Motor ability of young children from the viewpoint of a national survey in 2008. Sci. Phys. Educ..

[50] Guo H, Kim H (2022). Longitudinal changes in lifestyle behaviors and physical fitness of Japanese preschoolers during the COVID-19 pandemic: Results from a 7-year longitudinal study. PeerJ.

[51] Aggio D, Smith L, Hamer M (2015). Effects of reallocating time in different activity intensities on health and fitness: A cross sectional study. Int. J. Behav. Nutr. Phys. Act..

[52] Chau JY (2017). Sitting ducks face chronic disease: An analysis of newspaper coverage of sedentary behaviour as a health issue in Australia 2000–2012. Health Promot. J. Austr..

[53] Santos R (2014). The independent associations of sedentary behaviour and physical activity on cardiorespiratory fitness. Br. J. Sports Med..

[54] Gennuso KP, Gangnon RE, Matthews CE, Thraen-borowski KM, Colbert LH (2013). Sedentary behavior, physical activity, and markers of health in older adults. Med. Sci. Sports Exerc..

[55] Keevil VL (2016). Objective sedentary time, moderate-to-vigorous physical activity, and physical capability in a British cohort. Med. Sci. Sports Exerc..

[56] Jefferis BJ (2015). Trajectories of objectively measured physical activity in free-living older men. Med. Sci. Sports Exerc..

[57] Contardo Ayala AM, Salmon J, Dunstan DW, Arundell L, Timperio A (2022). Does light-intensity physical activity moderate the relationship between sitting time and adiposity markers in adolescents?. J. Sport Health Sci..

[58] Tortella P, Haga M, Loras H, Sigmundsson H, Fumagalli G (2016). Motor skill development in Italian pre-school children induced by structured activities in a specific playground. PLoS ONE.

[59] Beets MW (2016). The theory of expanded, extended, and enhanced opportunities for youth physical activity promotion. Int. J. Behav. Nutr. Phys. Act..

[60] Comte M (2013). Patterns of weekday and weekend physical activity in youth in 2 Canadian provinces. Appl. Physiol. Nutr. Metab..

[61] Vander Ploeg KA (2013). The importance of parental beliefs and support for pedometer-measured physical activity on school days and weekend days among Canadian children. BMC Public Health.

[62] McMinn AM, Griffin SJ, Jones AP, van Sluijs EM (2012). Family and home influences on children’s after-school and weekend physical activity. Eur. J. Public Health.

[63] Wang Q (2021). Associations among outdoor playtime, screen time, and environmental factors in Japanese preschoolers: The ‘eat, be active, and sleep well’ study. Sustainability.

[64] Wang Q, Ma J, Maehashi A, Kim H (2020). The associations between outdoor playtime, screen-viewing time, and environmental factors in Chinese young children: The “eat, be active and sleep well” study. Int. J. Environ. Res. Public Health.

